# Irony Processing in Adults with ADHD: Evidence From Eye-Tracking and Executive Attention Tasks

**DOI:** 10.1177/10870547251333819

**Published:** 2025-04-25

**Authors:** Marianna Kyriacou, Cecilie Rummelhoff, Franziska Köder

**Affiliations:** 1 University of Oslo, Norway

**Keywords:** adult ADHD, linguistics, executive function, communication, individual differences

## Abstract

**Objective:**

ADHD is a neurodevelopmental disorder that impacts pragmatic communication abilities in children, including their understanding of verbal irony. This study aims to investigate whether adults with ADHD experience similar challenges in interpreting ironic statements, and to examine the role of executive attention abilities in accounting for any observed differences.

**Methods:**

52 adults with ADHD and 55 neurotypical controls participated in an eye-tracking experiment. They read stories that included either literal or ironic statements and answered targeted comprehension questions. We used measures of working memory and fluid intelligence as independent indices of executive attention.

**Results:**

The results showed that adults with ADHD were as accurate as the control group in comprehending irony. However, they experienced an additional processing cost, indicated by increased reading times for ironic statements. While fluid intelligence improved comprehension accuracy in the control group, it did not have the same effect for participants with ADHD. Importantly, higher working memory capacity in adults with ADHD was associated with faster processing times, making their irony processing comparable to that of the control group.

**Conclusion:**

Our findings underscore the subtle challenges adults with ADHD face in processing irony and highlight the crucial role of working memory in enhancing performance. These insights stress the importance of considering individual cognitive capacities and their interaction with ADHD symptoms to better understand how ADHD impacts pragmatic abilities in adulthood.

## Introduction

ADHD is the most common neurodevelopmental disorder which is dramatically on the rise in adults, affecting approximately 2.5% to 6.8% worldwide (Polanczyk et al., 2014; Song et al., 2021). In both children and adults, ADHD is associated with neuropsychological disfunctions in areas of cognition, and especially in executive attention or attention control (Salomone et al., 2020), a central cognitive system responsible for self-control (Broadway et al., 2010), emotional regulation (Schmeichel & Demaree, 2010), and task engagement in daily life (Botvinick et al., 2004; Miller & Cohen, 2001). ADHD in children has also been linked to challenges with language and communication (Korrel et al., 2017), including difficulties with pragmatic language, such as understanding irony and sarcasm (Caillies et al., 2014; Ludlow et al., 2017; Silva et al., 2024). Research investigating the pragmatic abilities of adults with ADHD is, however, still scarce (see, e.g., Nilsen et al., 2013; Segal et al., 2015), while research on irony processing by this population is, to our knowledge, non-existent to date. Furthermore, it is still unclear what role executive attention abilities play in the comprehension and processing of irony in general, and especially in this clinical population where variability in executive attention abilities is considerable. The current study aims to address these research gaps by investigating whether adults with ADHD face challenges in understanding or processing ironic meanings in written texts, and whether individual differences in executive attention—more specifically in working memory and fluid intelligence—influence irony comprehension and processing.

### The Processing of Irony by Neurotypical Individuals

Everyday communication is laden with nonliteral language uses, an example of which is the use of irony. When for instance someone describes the weather as “lovely,” despite it being unfavorable, the listener must recognize the discrepancy between the literal meaning of the statement and the actual context. Subsequently, the listener needs to infer the speaker’s communicative intention (e.g., expressing frustration over their unmet expectations for a pleasant weather), potentially by entertaining second-order meta-representations as irony arguably communicates a thought about a thought, attributed to either one’s own prior self, another individual, or a general norm or expectation (Clark & Gerrig, 1984; Wilson & Sperber, 2012). Irony has a broad spectrum of communicative functions, including humor, criticism, emotion management, and politeness. Importantly, irony also plays a pivotal role in fostering in-group solidarity (Attardo, 2000; Gibbs, 2000). Consequently, an inability to fully engage in this social dynamic of irony may have adverse effects on an individual’s social relationships, both professionally and personally, potentially impacting their emotional well-being over time.

Psycholinguistic research typically demonstrates a processing cost for irony compared to literal language, which can manifest as increased reading times and/or regression probability (i.e., look-backs) in reading studies (e.g., Au-Yeung et al., 2015; Filik et al., 2014, 2018; Filik & Moxey, 2010; Kaakinen et al., 2014; Olkoniemi et al., 2016, 2019), as amplified N400/P600 effects in ERP studies (Cornejol et al., 2007; Filik et al., 2014; Regel et al., 2010, 2011; Spotorno et al., 2013), and as differential brain activity with respect to both locality and magnitude in fMRI studies (Akimoto et al., 2014; Bosco et al., 2017; Obert et al., 2016; Shibata et al., 2010; Spotorno et al., 2012). Studies incorporating explicit comprehension measures (i.e., questions targeting the interpretation of ironic versus literal statements), equally show reduced accuracy and increased response times for irony (e.g., Deliens et al., 2018; Kaakinen et al., 2014; Olkoniemi et al., 2016; Olkoniemi, Johander, et al., 2019; Olkoniemi, Strömberg, et al., 2019).

Notably, while factors such as default phrasal interpretations, the strength and kind of contextual constraints, cultural expectations, as well as individual differences in, for instance, mentalizing skills and emotion understanding can facilitate the processing of irony (e.g., Filippova & Astington, 2008; Gibbs, 1994; Giora, 2003; Jacob et al., 2016; Katz et al., 2004; Katz & Ferretti, 2001; Nicholson et al., 2013; Nilsen et al., 2011; Pexman, 2008; Ronderos et al., 2024; Spotorno & Noveck, 2014), it is evident that irony comprehension often poses challenges to the language user and is also acquired later than other types of figurative language such as metaphor and metonymy (Falkum & Köder, 2020).

The source of this irony processing cost cannot be clearly pinpointed, as it may be attributable to (literal vs. ironic) meaning activation and disambiguation processes, and/or to the availability of (enough) contextual cues and phrasal constraints, as well as individual differences in (among other) metarepresentational and inferential skills. Arguably, the processing of irony at the very least involves the concurrent manipulation of multiple sources of information, which in often subtle ways, point to an ironic meaning.

Because of this, it is likely that irony comprehension taxes the cognitive system more heavily than literal language, and as such, it may be directly affected by cognitive abilities. Executive attention, or attention control, is central to models of higher-order cognition (e.g., Atkinson & Shiffrin, 1968; Baddeley, 1998; Draheim et al., 2021; Posner & DiGirolamo, 1998; Shipstead et al., 2016), and relates to one’s ability to focus and perform on a task by regulating thoughts and behavior. As executive attention abilities vary among individuals, this may influence, for example, how well and how fast irony is detected and understood.

### The Role of Executive Attention in Irony Processing by Neurotypical Individuals

Within a domain-general framework, executive attention is mediated by working memory and fluid intelligence (Burgoyne & Engle, 2020; Engle, 2002, 2018; Shipstead et al., 2016). Working memory, a multi-component cognitive construct with a limited capacity (Baddeley, 2010; Cowan, 2008), is responsible for managing information manipulation, storage, recall, and processing over a short period of time (Baddeley & Hitch, 1974; Baron, 2004; Chai et al., 2018). It involves the ability to concurrently hold and manipulate information in memory to accomplish a task (Chai et al., 2018), and plays a crucial role in sustaining attention to task during performance by filtering out distractions from both external sources, like surrounding noises, and internal ones, like irrelevant thoughts (Engle, 2018). Fluid intelligence, on the other hand, relates to problem-solving skills, and in particular, the ability to find solutions to novel problems and to reason with novel information (Cattell, 1943; Horn & Cattell, 1966). It enables disengaging from incorrect solutions, thus allowing attentional resources to be reallocated elsewhere to enhance task performance (Burgoyne & Engle, 2020; Engle, 2002, 2018; Shipstead et al., 2016). Importantly, both constructs rely on the same attention control system: working memory sustains attention, while fluid intelligence disengages it for effective processing.

From a theoretical perspective, greater working memory could ease the processing of irony, as a larger processing capacity and an enhanced ability to focus could better accommodate multiple concurrent pieces of information (e.g., contextual cues, speaker intentions, and alternative meanings) until meaning disambiguation and selection has been finalized. On the other hand, if we conceptualize irony as a problem to be solved, greater fluid intelligence may facilitate processing by offering better problem-solving skills and possibly more efficient disengagement of the nontarget literal meaning of an ironic utterance. Arguably, these constructs may be mobilized to a greater or lesser degree depending on how taxing an ironic statement is. For example, it is possible that default ironic statements (e.g., *He’s not the smartest student in the class*; (Giora et al., 2015) are immediately recognized as such, thus leading to a significantly lower cognitive load, minimizing as a result the involvement of working memory or fluid intelligence.

While the exact role of executive attention in pragmatic language competence is not fully understood, a few studies suggest a potentially significant role. Regarding the processing of verbal irony during reading, increased working memory capacity has been associated with *increased* reading times for ironic as opposed to literal statements (Kaakinen et al., 2014; Olkoniemi et al., 2016). This has been taken to reflect earlier irony detection and therefore earlier engagement in irony processing. Lower working memory capacity, on the other hand, has been linked to increased regressions to ironic as opposed to literal statements, indicating reanalysis and/or problematic meaning integration in the case of irony (Olkoniemi et al., 2016; Olkoniemi, Johander, et al., 2019). A more recent study, however, reports only an *indirect* role of working memory, with fluid intelligence acting as the mediator and the main significant predictor (Kyriacou & Köder, 2025). Specifically, higher fluid intelligence was found to improve both accuracy and response times to inference questions tapping into the comprehension of ironic statements, while lower fluid intelligence resulted in increased regressions to the text region containing disambiguating information. This suggests that readers with lower fluid intelligence experienced specific problems in understanding irony and often resorted to reanalysis by going back to reread pertinent contextual cues. Some other findings have further shown positive correlations between fluid intelligence and the ability to detect ironic praise in aptitude tests (i.e., negative statements expressing positive attitudes; Bruntsch & Ruch, 2017).

It is possible that where working memory is sufficient (as can be assumed for the neurotypical individuals in Kyriacou and Köder’s (2025) study), higher fluid intelligence may provide additional processing advantages to the processing of irony. This is because the adequate processing capacity of working memory can better accommodate problem-solving operations. Conversely, along with a limited working memory capacity, the ability to mentally manipulate information, including the ability to consider alternative hypotheses or solutions to a problem can also be hampered. In other words, problem-solving operations associated with fluid intelligence also depend on working memory’s processing capacity, and as such, fluid intelligence effects might be subject to working memory limitations (for a lengthier discussion on this see also Jastrzębski et al., 2018). This raises further interesting questions about the existence of a minimum working memory threshold for fluid intelligence effects to emerge in irony processing, and whether this has implications for populations with suboptimal working memory capacity.

### The Processing of Irony by Neurodiverse Individuals

Certain groups face additional challenges when processing irony. Young children (Falkum & Köder, 2020), as well as second-language users (Ellis et al., 2021), for instance, find irony particularly difficult to understand, subject to developmental maturity and second language proficiency. Irony comprehension is also affected by certain neurodevelopmental and other mental disorders. For instance, difficulties with irony comprehension are well documented in individuals with autism spectrum disorder (ASD; e.g., Caillies et al., 2014; Chahboun et al., 2021; Martin & McDonald, 2005; Wang et al., 2006), and in those with schizophrenia (Adamczyk et al., 2024). ADHD has also been linked to problems with nonliteral language comprehension, although most of the data to date come from studies with children. Specifically, previous research has demonstrated that children with ADHD struggle with metaphors (e.g., *he is a lion*, meaning ‘he is brave’) and idiomatic expressions (e.g., *to be short with someone*, meaning ‘to speak rudely to someone without saying much’; Adachi et al., 2004; Bignell & Cain, 2007), as well as irony (Caillies et al., 2014; Carruthers et al., 2022; Ludlow et al., 2017; Silva et al., 2024). In children, pragmatic problems remain even after controlling for general language ability (Staikova et al., 2013), suggesting that the source of pragmatic deficits in ADHD is not lower linguistic ability. Consequently, questions arise as to whether pragmatic deficits in ADHD—particularly difficulties with irony—persist into adulthood along with the disorder itself (Fayyad et al., 2007; Kessler et al., 2006).

Despite being an under-researched group with respect to pragmatic abilities, adults with ADHD also seem to exhibit some pragmatic language deficits, in line with what has been observed for children with the same disorder. One study shows that adults with ADHD are less efficient than neurotypical individuals at processing metaphors as opposed to literal phrases (Segal et al., 2015). More recently, adults with ADHD were found to perform worse than controls in pragmatic comprehension and production tasks, especially when required to produce verbal explanations of figurative language uses, such as idioms, metaphors, and common proverbs (Even-Simkin, 2024). Regarding irony comprehension, evidence to date is limited to self-report data, which indicate that adults with ADHD seem to experience more difficulties with irony understanding than controls (Köder et al., 2024).

Thus, the relevant findings from children with ADHD, as well as the emerging evidence from adults point to pragmatic and figurative language difficulties, highlighting the possibility of irony being a stumbling block also for adults with ADHD. Moreover, as irony likely places considerable demands on the cognitive system, it might be more costly for individuals with ADHD who also experience more severe impairments in executive attention.

### Attention Control and Irony Processing in Individuals with ADHD

ADHD in both children and adults is associated with deficits in working memory and fluid intelligence (for reviews see: Anderson et al., 2002; Ramos et al., 2020; Shallice et al., 2002), although certain aspects of attention control and in particular working memory and attention can be improved with ADHD psychostimulant medication (Mckenzie et al., 2022). These deficits may be neuroanatomical by nature, as structural differences in the brain have been observed in individuals with ADHD relative to controls, in regions tied to these cognitive functions (Bush et al., 2005; Seidman et al., 2006; Valera et al., 2007).

Observations from behavioral studies paint a similar picture, in showing that adults with ADHD perform worse than controls on standardized fluid intelligence (Dige & Wik, 2005; Goodwin et al., 2011) and working memory tests (Alderson et al., 2013; Schweitzer et al., 2006). With respect to working memory, limitations are assumed to stem from inadequate attention control (e.g., the inability to filter out irrelevant information/distractors) which overloads working memory and, as a result, limits its processing capacity (Burgess et al., 2010). Alternatively, it has been argued that the issue lies in an inability to efficiently administer attention and working memory resources where needed. Consequently, the encoding of incoming information is suboptimal (Ortega et al., 2020), leading to less effective information maintenance and manipulation in the mind (Fosco et al., 2020). Regarding the observational findings on fluid intelligence, it is worth pointing out that the relevant studies were conducted with individuals who had additional psychiatric or neurological conditions or were recruited from correctional facilities. Therefore, these findings might not be representative of problem-solving skills in individuals with ADHD at large. Furthermore, some findings suggest that fluid intelligence deficits in ADHD may be more severe with a predominant hyperactive (as opposed to inattentive) ADHD manifestation, or they may only emerge when working memory problems are also present (Brydges et al., 2017, 2021). This relates to our earlier argument about the importance of having a sufficiently developed working memory for fluid intelligence to exert (additional) effects on processing.

Taken together, these findings suggest possible differences in working memory capacity and fluid intelligence between individuals with ADHD and neurotypical individuals. Compared to neurotypical individuals therefore, adults with ADHD may struggle more when processing irony due to a more limited working memory capacity and less efficient problem-solving skills. It is also possible that fluid intelligence may only affect processing if working memory is good enough to enable the smooth functioning of problem-solving operations. Otherwise, if working memory is inadequate, fluid intelligence may not emerge as a significant predictor.

### The Present Study

Building upon the previous discussion, additional research is necessary to uncover the intricate relationship between executive attention and irony processing, especially in adults with ADHD, a disorder that directly impacts executive attention skills. The present study primarily aims to examine whether individuals with ADHD continue to struggle with irony in adult life and whether working memory capacity and fluid intelligence influences their irony comprehension and processing.

To address these aims, we recruited adult participants officially diagnosed with ADHD, and adopted Kyriacou and Köder’s (2025) design including an eye-tracking reading task, two executive attention measures, and an ADHD screening questionnaire. The researchers’ published data on neurotypical participants served as the control group for the current study. All study materials, analysis code, datasets, and Supplemental Materials can be accessed at https://osf.io/zygmd/?view_only=70aee8d72cbe4cd189237a7453fb3b24. The preregistration of the study can be found at https://osf.io/hr2fd.

We put forward the following hypotheses:

1. Adults with ADHD may experience *specific* difficulties when processing irony. In reading, this could be reflected by prolonged reading times and higher regression probability for ironic as opposed to literal statements, and in lower accuracy in the comprehension task. Crucially, as irony can similarly induce a processing cost to neurotypical individuals, a specific irony cost for adults with ADHD should be significantly more accentuated in comparison. Notably, considering the high comorbidity of ADHD with learning disorders such as dyslexia and reading disorder (e.g., Daley & Birchwood, 2010; Germanò et al., 2010; Parks et al., 2022; Sexton et al., 2012; Willcutt, 2018), a specific irony cost in reading, in adults with ADHD, should be independent of (any) general reading difficulties (e.g., slower overall reading pace; Stern et al., 2024).
2. Adults with ADHD and higher fluid intelligence may demonstrate improved accuracy and response time to inference questions targeting the interpretation of irony, whereas those with lower fluid intelligence may exhibit increased reanalysis of the context in the irony condition, lining up with the findings from neurotypical individuals reported in Kyriacou and Köder (2025).
3. Considering the potential deficits in working memory among individuals with ADHD, and the dependency of fluid intelligence on working memory capacity, working memory might also affect irony processing in adults with ADHD.

## Methods

### Participants

We recruited in total 113 adult participants with Norwegian as a first language and without known dyslexia or other learning disorders. Due to technical failure of the working memory task, we had to exclude three participants whose data were not saved for this task. Another three participants were excluded due to reporting a comorbid ASD diagnosis, a disorder known to cause irony comprehension difficulties (Fuchs, 2023; Mention et al., 2024), thus leaving 107 participants. There were two groups of participants that were age-matched on a group level (between 18 and 35 years old) minimizing confounding factors related to cognitive development or decline: typically-developed (TD) readers without ADHD (*N* = 55, mean age = 23.11, *SD* = 3.92, females = 37, males = 18), and readers with an official ADHD diagnosis (*N* = 52; mean age = 27.84, *SD* = 5.58, females = 38, males = 14). The data from the TD group have already been published in Kyriacou and Köder (2025). Participants signed an informed consent form prior to the start of the session and received a gift card for their participation. Testing took place at the Socio-Cognitive Laboratory at the University of Oslo.

The study was approved by the Norwegian Agency for Shared Services in Education and Research Sikt (reference number 478374), and the Regional Ethics Committee (reference number 578166), the national organization regulating the collection and manipulation of health data in Norway. Participants with ADHD were invited to briefly discontinue their ADHD pharmacological treatment (if medicated for the disorder) for the last 24 hr leading to the experimental session. There are currently no standard guidelines regarding the duration of medication interruption required to capture “raw” ADHD effects in behavioral experiments, with prior studies ranging between 12, 24, and 48 hr (Alvarado et al., 2011; Friedman et al., 2017; Madjar et al., 2020; Miranda et al., 2017). We opted for a 24 hr interruption as the effects of most ADHD medications, including those with long-acting effects, would not typically last longer than this timeframe (Jansen, 2018). Of the 52 participants with ADHD, 12 did not take ADHD medication in general and were therefore unmedicated ahead of the experiment, 25 typically took ADHD medication but were unmedicated ahead of the experiment, and 10 typically took ADHD medication and were also medicated on the day of the experiment. The remaining five participants with ADHD did not report on their medication status. Importantly, separate statistical analyses focusing only on individuals with ADHD revealed that medication status did not significantly influence reading patterns, or performance on the cognitive tasks. For this reason, the main analyses reported below include all participants with ADHD regardless of medication status. An additional analysis excluding medicated participants with ADHD is included in the Supplemental Materials.

### Materials and Procedure

#### Eye-Tracking Reading Task

Participants were required to (silently) read stories on a computer monitor, while their eye-movements were recorded by an SR EyeLink 1000+ desktop-mount eye-tracker (SR Research, Ontario, Canada), with a sampling rate of 1,000 Hz. Each story had two versions, one where a phrase (henceforth referred to as the target phrase) was intended literally, and one where the target phrase was intended ironically. The target phrases were the same across the two versions. There were in total 24 story pairs (*N* = 48, mean length = 721.24 characters, *SD* = 47.56). Each story comprised three regions of interest, always presented in the following order: (a) the *context region* which contained disambiguating information regarding the intended meaning of the target phrase, (b) the *target phrase region*, and (c) the *spillover region* which contained any remaining text after the target phrase region. We reproduce Kyriacou and Köder’s (2025) stimuli example in Table 1, translated to English. The original materials were developed in Norwegian. The experimental stories underwent a series of norming procedures to ensure that target phrases were interpreted as intended in their corresponding context (i.e., literally vs. ironically), and that the stories were equally natural in both conditions (for more details see Kyriacou & Köder, 2025). In addition to the experimental stories, there were 12 additional filler stories of a similar length and structure.

**Table 1.** Example Stimuli Stories, General Content Question and Inference Question Across Phrase Type (Ironic and Literal), Translated to English, Reproduced From Kyriacou and Köder (2025).

	Phrase type	
Region	Ironic	Literal
Context region	Sarah and Jack have different approaches to life. Jack is a stickler for punctuality, he is always super organized, and well-prepared in advance; something that Sarah finds quite irritating at times. When they fly together, Jack always insists that they get to the airport at least 4 hr ahead of their departure to allow for any unexpected delays. Yesterday, they were flying out to the Fiji Islands for holidays. When they got to the airport, they found out that their flight had been **postponed for 3 hr** due to scheduling changes, although they hadn’t been informed by the airlines.	Sarah and Jack have different approaches to life. Jack is a stickler for punctuality, he is always super organized, and well-prepared in advance; something that Sarah finds quite irritating at times. When they fly together, Jack always insists that they get to the airport at least 4 hr ahead of their departure to allow for any unexpected delays. Yesterday, they were flying out to the Fiji Islands for holidays. When they got to the airport, they found out that their flight had been **brought forward by 3 hr** due to scheduling changes, although they hadn’t been informed by the airlines.
Target phrase region	**“Well, good thing we got here early!”,**	**“Well, good thing we got here early!”,**
Spillover region	commented Sarah. Jack mumbled something along the lines of, “They ought to have told us, really,” and went on to check his emails for any missed correspondence.	commented Sarah. Jack mumbled something along the lines of, “They ought to have told us, really,” and went on to check his emails for any missed correspondence.
General content question	Does Sarah always appreciate Jack’s organizing skills?	
Correct answer	No	No
Inference question	Was Sarah glad that she and Jack had arrived at the airport earlier?	
Correct answer	No	Yes

*Note*. Biasing contextual information in the context region, and the target phrases are presented in bold in the example above but were in no way demarcated for participants.

After reading each (experimental or filler) story, participants were sequentially presented with two YES/NO comprehension questions. Question 1 tapped into general content comprehension, while Question 2 tapped into the interpretation of the target phrases. Question 2 is henceforth referred to as inference question, since it required drawing inferences pertaining to the mental state (i.e., feelings/ beliefs/ intentions) of the character who uttered the target phrase. An example for each type of question is provided in Table 1. To answer the questions, participants pressed the designated keys on the keyboard corresponding to YES or NO. It was not possible to go back to the story once the comprehension task had started.

The stories were distributed across two counterbalanced lists, each containing 12 ironic and 12 literal phrases. Therefore, participants only read each experimental story in one of the two conditions (literal or irony), while the same 12 filler stories appeared in both lists. Participants were instructed to read the stories as quickly as possible without compromising comprehension, and to avoid unnecessary rereading. After reading each story, participants pressed ENTER to proceed to the comprehension task and then again to proceed to the next story. The presentation of the stories was randomized per participant, and each story was presented on a single screen, in black font (Courier New, size 16) over a white background, one at a time, using triple line spacing. Participants were seated in front of a computer monitor and a chin-and-forehead rest was used to minimize head movements. The eye-tracker was calibrated on a nine-point grid, drift correction preceded the presentation of each story, and recalibration was performed whenever necessary.

#### Working Memory Task

To estimate working memory capacity, participants were administered the Shortened Symmetry Span Task (SSPAN; Foster et al., 2015), a computerized task adapted to Norwegian at our lab. In this task, participants engage in both an irrelevant distractor task, known as the judgement task, and a target memory task. For the distractor task, participants are required to judge the symmetry of a geometrical pattern along the vertical axis, while for the memory task, participants need to recall which cell(s) within a blank matrix turn red and in which order. The distractor task alternates with the memory task for a varying number of sequences, and each time a single block within the matrix turns red. After this alternating sequence, participants are presented with a blank matrix and are required to click on the locations of the red cells in the order they appeared. The number (and location) of the red cells that need to be remembered varies from two to five across different trials. The task comprises three blocks, with a maximum of 14 points per block (42 in total). Participants’ performance was scored based on the total number of correct item recalls and score credit was also allocated to partially correct items (Conway et al., 2005). Working memory scores were calculated using the *englelab* package (Tsukahara, 2022) in R, version 4.3.2 (R Core Team, 2023). The analysis of the scores revealed no significant difference in working memory capacity between participants with ADHD (*M* = 26.92 [/42], *SD* = 8.15, range = 8–42) and TD participants (*M* = 29.05[/42], *SD* = 8.17, Range = 7–41; *t*(105 = −1.35, *p* = .18).

#### Fluid Intelligence Task

To estimate fluid intelligence, we used a digitized version of Raven’s Advanced Progressive Matrices (Pearson, 2015), a nonverbal task tapping into problem-solving abilities. The task comprises 23 items/problems, each consisting of a black-and-white geometric pattern with a missing piece. Participants are required to solve the problems by identifying which one out of the eight available options is the correct one. Due to time limitations, participants were given 10 min to solve as many problems as they could. Fluid intelligence scores were calculated based on the total number of items answered correctly and are reported in percentages. The analysis of the scores revealed no significant difference in fluid intelligence between participants with ADHD (*M* = 33.87 [/100], *SD* = 12.52, Range = 13.04–65.21) and TD participants (*M* = 32.65[/100], *SD* = 14.78, Range = 4.35–65.21; *t*(104) = 0.46, *p* = .65).

#### Adult ADHD Self-Report Scale

As an independent measure of inattention and hyperactivity/impulsivity, both participants with and without ADHD completed Kessler et al.’s (2005) Adult ADHD Self-Report Scale-v1.1 (henceforth referred to as ASRS), a tool used for screening of ADHD symptoms. The self-report scale consists of 18 questions, divided in two parts: Part A (*N* = 6) and Part B (*N* = 12). The questions are designed to tap into behavioral symptoms of inattention (e.g., “How often do you have trouble wrapping up the final details of a project, once the challenging parts have been done?”) and hyperactivity/impulsivity (e.g., “How often do you feel overly active and compelled to do things, like you were driven by a motor?”). When completing the Scale, individuals are advised to answer the questions in relation to how they have felt and conducted themselves over the past 6 months. Answers are provided on a 5-point scale (“never,” “rarely,” “sometimes,” “often,” and “very often”), and are scored with 0 or 1 on an item-by-item basis (for further details on the scoring system see Kessler et al., 2005). A score of 4 or higher (maximum 6) in Part A prompts a referral to a specialist, as Part A questions have been proven to be the most predictive of ADHD. As expected, readers with an official ADHD diagnosis scored on average above 4 in Part A (*M* = 4.70, *SD* = 1.24, Range = 1–6), while TD readers scored on average below 4 (*M* = 2.67, *SD* = 1.56, Range = 0–6). The difference between groups was statistically significant (*t*(102) = −7.417, *p* < .001), confirming the reliability of ASRS in distinguishing between clinical and nonclinical groups. This also confirmed that our participants were representative of their respective group. To enable group comparisons, we used Group as the fixed variable of interest instead of ASRS scores.

Part B of the Scale is intended to provide more information regarding specific inattention/hyperactivity/impulsivity symptoms. To further explore the attentional profile of our readers, we collated the scores of all attention-related questions (i.e., Questions 1–4 and 7–11) and those of hyperactivity/impulsivity-related questions (i.e., Questions 5–6, and 12–18), from both Part A and B, and plotted them across Groups. A Pearson’s correlation test indicated a positive correlation between inattention and hyperactivity/impulsivity symptoms, with *r* = .68 and *t*(105) = 9.29, *p* < .001, which remained significant even when performing separate tests per Group (ADHD: *r* = .55; *t*(50) = 4.61, *p* < .001 and TD: *r* = .52; *t*(53) = 4.46, *p* < .001). This pattern suggests that more (or less) severe inattention problems were accompanied by equally more or less severe hyperactivity/impulsivity symptoms (and vice versa). Therefore, while in Norway ADHD diagnosis does not consistently distinguish between different presentations (i.e., predominantly hyperactive, predominantly inattentive, or combined), these data suggest that our pool of participants with ADHD experienced symptoms related to both inattention and hyperactivity/impulsivity to a similar extent.

#### The Experimental Session

Each experimental session began with the reading task and was followed by the working memory and fluid intelligence tasks, the order of which was counterbalanced across participants. The ASRS Scale was administered last. The entire experimental procedure lasted between 60 and 90 min, depending on individual reading speed.

## Results

Data were analyzed in R, version 4.3.2 (R Core Team, 2023), using mixed effects models and the *lme4* package, version 1.1-35.1 (Bates et al., 2014).

### The Reading Task

For the target phrase region, we examined *first pass reading time* (i.e., the duration of all fixations during first pass reading only), *first pass gaze duration* (i.e., the duration of all fixations *and* refixations during first pass reading), *total reading time* (i.e., the duration of all fixations and refixations), and regression likelihood (i.e., the likelihood of the region to be revisited from subsequent parts of the text). For the context region, we examined *regression likelihood*, and for the spillover region *first fixation duration* (i.e., the duration of the first fixation occurring in the region). Reading time measures were not analyzed for the context region, as these measures would be susceptible to linguistic differences present across the two conditions. Regression probability on the other hand is typically associated with reanalysis and global meaning integration, making it less susceptible to low-level linguistic differences. In addition to the reading measures, we also examined response *accuracy* (1,0) and *response time* to the inference questions of the comprehension task, which targeted the interpretation of the literal or ironic target phrases.

Response accuracy to the general comprehension questions was high across both groups (ADHD: *M* = .86, *SD* = 0.34, TD: *M* = 0.87, *SD* = 0.33), indicating that participants were attentive to the task and that overall reading comprehension was comparable across groups. Outliers were removed case-by-case after graphically exploring the data split by measure and Region of Interest. The removal of outliers led to a range of 0% to 1.17% of data loss across all measures and Regions of Interest. Of note, 16% of the data on accuracy on inference questions were unusable due to a programming error in the original experiment (see Kyriacou & Köder, 2025, for further details).

Phrase Type (irony vs. literal), Group (TD vs. ADHD), working memory score, fluid intelligence score, and Trial Index (i.e., stimuli presentation order) were specified as fixed effects in the models. Trial Index was included to account for learning/fatigue effects as the experiment progressed. We further specified interactions between Phrase Type, Group, and fluid intelligence/working memory scores. Phrase Type and Group were effect-coded with the literal condition and TD group respectively set as the reference levels. All remaining factors were centered and scaled. Working memory and fluid intelligence scores exhibited a mild but significant, positive correlation (*r* = .27; *p* = .005), without, however, evidence of multicollinearity in the models (all VIF values < 2). Both scores were therefore included as predictors in the models. Maximal models were specified with by-participant and by-item correlated random intercepts and slopes for Phrase Type and Trial Index, as well as random intercepts of fluid intelligence and working memory scores in interaction with Phrase Type and Trial Index for items. Reading and response time measures were log-transformed and analyzed via linear models, while binary measures (regression probability and accuracy) were analyzed using logistic regressions. Fixation durations shorter than 80 ms were removed or merged with the largest nearest fixation for a distance up to 0.5° of visual angle (Eskenazi, 2024). All maximal models led to singular fits and/or convergence issues even when using optimization. Final models were therefore selected by reducing the random effect structure until no more convergence or singular fit issues arose. We simplified the random effect structure first by removing interactions between random effects, and subsequently by removing effects whose variance estimates were (too close to) 0 or 1. The outputs of maximal models are available to view in Supplemental Materials while those of the final models are presented in the Appendix. Below we only report significant effects for brevity. Significant effects arising from Group, fluid intelligence, working memory, and any interactions among these variables were confirmed by comparing the final model to null models lacking the relevant fixed effect. Model comparisons between final and null models were performed using *anova()* in R.

#### Phrase Type, Group, and Trial Index effects

Phrase Type was significant in first pass gaze duration (β = .03, *SE* = 0.01, *t* = 5.12, *p* < .001) and total reading time (β = .07, *SE* = 0.01, *t* = 5.57, *p* < .001) of the target phrase region, with ironic phrases eliciting longer reading times than literal ones. Additionally, it was significant in regression probability to both the target phrase region (β = .24, *SE* = 0.05, *z* = 4.77, *p* < .001) and context region (β = .15, *SE* = 0.05, *z* = 3.01, *p* = .003), with regression probability increasing in the irony compared to the literal condition. Phrase Type was also significant in first fixation duration of the spillover region (β = .02, *SE* = 0.01, *t* = 2.73, *p* = .006), whereby fixation durations were significantly longer in the irony compared to the literal condition. Furthermore, irony led to a significant decrease in accuracy (β = −.32, *SE* = 0.07, *z* = −4.57, *p* < .001), and a significant increase in response time (β = .05, *SE* = 0.01, *t* = 3.37, *p* = .001) to the inference questions of the comprehension task compared to the literal condition. This means that across different measures and regions of interest, ironic phrases elicited longer reading times, more regressions, longer response times as well as lower comprehension accuracy scores compared to literal phrases.

Group was significant in first pass gaze duration (β = .08, *SE* = 0.03, *t* = 2.61, *p* = .009) and total reading time (β = .09, *SE* = 0.03, *t* = 2.90, *p* = .004) of the target phrase region and in regression likelihood to context region (β = .57, *SE* = 0.13, *z* = 4.41, *p* < .001). Readers with ADHD produced longer reading times than TD readers and were more likely to revisit the context region. Additionally, there was a significant interaction between Phrase Type and Group in the same measures: that is, in first pass gaze duration (β = .01, *SE* = 0.01, *t* = 2.14, *p* = .03) and total reading time of the target phrase region (β = .03, *SE* = 0.01, *t* = 4.38, *p* < .001). Readers with ADHD were particularly slower at reading ironic as opposed to literal phrases compared to TD readers. For means across measures and regions of interest see Table 2.

**Table 2.** Means Across Reading and Comprehension Measures and Regions of Interest, Split by Phrase Type, and Group by Phrase Type.

	Phrase type							
	Literal		Irony					
Measures	*M*	*SE*	*M*	*SE*				
Target phrase region								
First pass reading time	847.01	16.35	892.63	17.42				
First pass gaze duration	**1,006.31**	16.72	**1,066.20**	16.95				
Total reading time	**1,166.20**	20.33	**1,338.08**	23.36				
Regression probability	**0.21**	0.01	**0.28**	0.01				
Context region								
Regression probability	**0.64**	0.01	**0.70**	0.01				
Spillover region								
First fixation duration	**237.59**	2.26	**248.14**	2.69				
Comprehension task								
Accuracy to inference question	**0.83**	0.01	**0.75**	0.01				
Response time to inference question	**3,523.05**	57.11	**3,932.13**	68.66				
	Group by phrase type							
	ADHD				TD			
	Literal		Irony		Literal		Irony	
Measures	*M*	*SE*	*M*	*SE*	*M*	*SE*	*M*	*SE*
Target phrase region								
First pass reading time	849.02	24.98	936.64	28.52	845.12	21.35	851.02	20.42
First pass gaze duration	**1,070.58**	24.88	**1,168.30**	26.64	**945.83**	22.24	**969.66**	20.60
Total reading time	**1,237.04**	30.13	**1,499.63**	36.18	**1,099.56**	27.22	**1,185.34**	28.69
Regression probability	0.22	0.02	0.30	0.02	0.19	0.02	0.26	0.02
Context region								
Regression probability	0.75	0.02	0.79	0.02	0.54	0.02	0.61	0.02
Spillover region								
First fixation duration	233.39	3.62	244.80	4.20	241.55	2.76	251.30	3.40
Comprehension task								
Accuracy to inference question	0.80	0.02	0.75	0.02	0.89	0.02	0.75	0.02
Response time to inference question	3,548.47	79.53	4,043.34	108.63	3,499.01	81.83	3,826.99	85.30

*Note*. Reading and response time means are reported in milliseconds, regression probability and accuracy means as probability from 0 to 1. Bold values denote significant differences between Phrase Type or Group by Phrase Type respectively.

There were no significant effects in first pass reading time of the target phrase region. Trial Index was significant in most models (for coefficients see model outputs), whereby as experience with the experiment accumulated, reading times, response times, and regression probability reduced, while comprehension scores increased, indicating a learning/facilitation effect for both groups. Taken together, these findings suggest that overall, readers with ADHD exhibited longer reading times and more regressions than TD readers, and crucially, they seemed to have particular difficulty in processing irony compared to TD readers.

#### Fluid Intelligence

**Figure 1.**
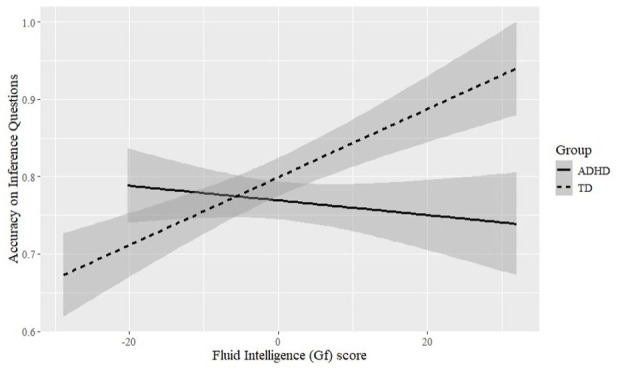
Accuracy on inference questions across Group as a function of (centered) fluid intelligence score.

There were no simple effects of fluid intelligence in any measure investigated. There was however an interaction between Group and fluid intelligence score in accuracy to inference questions (β = −.02, *SE* = 0.02, *z* = −3.05, *p* = .002), with TD readers showing significant improvement in accuracy as fluid intelligence score increased. In contrast, the accuracy of readers with ADHD was unaffected by fluid intelligence score (Figure 1). There was also a significant three-way interaction between Phrase Type, Group, and fluid intelligence in regression probability to context region (β = .01, *SE* = 0.00, *z* = 2.23, *p* = .02). A visual inspection of the data showed that this interaction was again driven by TD readers, whereby, TD readers with lower fluid intelligence were more likely to regress to the context region in the irony rather than the literal condition. The latter interaction has already been thoroughly examined in Kyriacou and Köder (2025) and will not be explored here further as it does not offer new insight regarding participants with ADHD. It appears, therefore, that fluid intelligence did not play a significant role in (irony) processing for readers with ADHD, nor did it bestow any advantages in comprehension accuracy for this Group.

#### Working Memory

**Figure 2.**
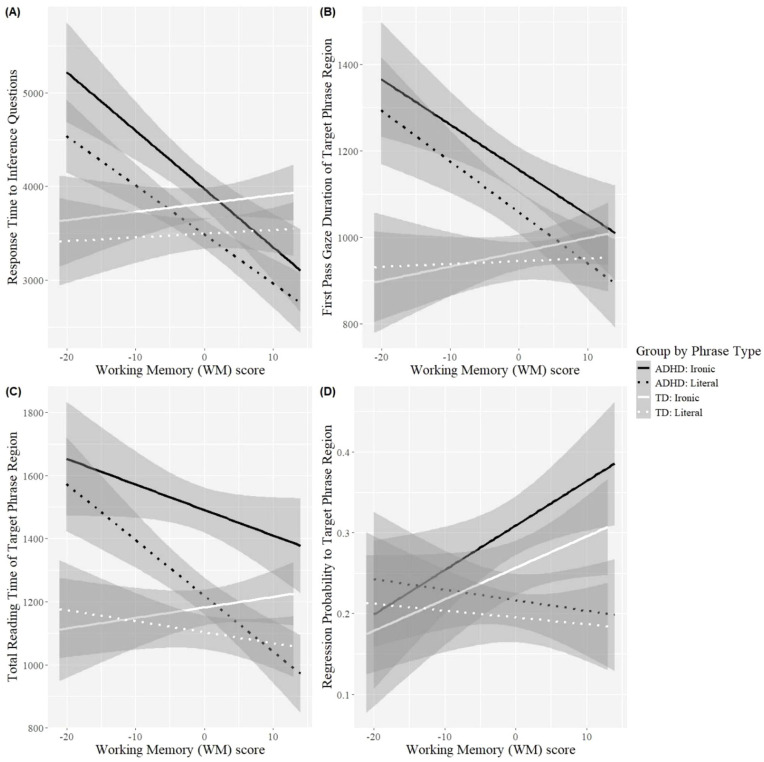
Graphs illustrating interactions between (centered) working memory score and Group (ADHD and TD) by Phrase Type (Ironic and Literal), in response time to inference questions (Panel A), first pass gaze duration of the Target Phrase Region (Panel B), total reading time of the Target Phrase Region (Panel C), and regression probability to the Target Phrase Region (Panel D).

There was a simple working memory effect in response time to inference questions of the comprehension task (β = −.01, *SE* = 0.00, *t* = −2.18, *p* = .03), with higher working memory leading to faster response times. A further interaction between Group and working memory in the same measure revealed that the effect was driven by readers with ADHD (β = −.01, *SE* = 0.01, *t* = −3.52, *p* < .001), as response times for TD readers were largely unaffected by working memory score (Figure 2, panel A). The interaction showed that readers with ADHD and lower working memory had slower response times relative to TD readers with lower working memory, while readers with ADHD and higher working memory were as fast as (or even slightly faster than) TD readers with higher working memory.

Group and working memory also interacted in first pass gaze duration (β = −.01, *SE* = 0.00, *t* = −2.43, *p* = .01) and total reading time (β = −.01, *SE* = 0.00, *t* = −2.07, *p* = .04) of the target phrase region. In total reading time, there was also a significant interaction between Phrase Type and working memory (β = .00, *SE* = 0.00, *t* = 2.04, *p* = .04). Readers with ADHD and lower working memory read overall significantly more slowly than TD readers with lower working memory, while readers with ADHD and higher working memory read at the same rate as TD readers with higher working memory (Figure 2, panels B and C). Furthermore, while greater working memory reduced total reading times for both ironic and literal phrases in readers with ADHD, ironic phrases still elicited significantly longer reading times relative to literal ones. Importantly, however, the total reading time of ironic phrases in readers with ADHD and higher working memory did not reliably differ from that of TD readers with higher working memory. Finally, there was a significant interaction between Phrase Type and working memory score in regression probability to the target phrase region (β = .02, *SE* = 0.01, *z* = 3.03, *p* = .002). Both Groups demonstrated increased regression probability to the Target Phrase Region in the irony as opposed to the literal condition when working memory was *higher* (Figure 2, Panel D). No differences in regression probability were noted across Phrase Type or Group when working memory was lower. Overall, the findings from reading time measures suggest that greater working memory in individuals with ADHD may compensate for some processing drawbacks potentially associated with the disorder (e.g., slower reading and response times), and may reduce the processing gap between ADHD and TD readers with regard to the processing of irony. Furthermore, greater working memory may lead to differential or strategic processing of irony in both readers with and without ADHD, as implied by the regression patterns.

## Discussion

This study aimed to examine the processing and comprehension of irony by adults with ADHD, and to explore how individual differences in executive attention, specifically in working memory and fluid intelligence, might affect processing relative to TD adults. To this end, we conducted an eye-tracking reading experiment to analyze the eye movements of participants with ADHD as they read stories containing ironic or literal phrases. We implemented inference questions as an explicit measure of comprehension, and a fluid intelligence and working memory task as independent indices of executive attention skills. We compared our findings to those on age-matched TD individuals (Kyriacou & Köder, 2025). In line with our first hypothesis, we found a specific irony processing cost in adults with ADHD, which however, did not affect explicit comprehension. Furthermore, in line with our third hypothesis, irony processing was modulated by working memory in individuals with ADHD, with greater working memory capacity making irony (and general) processing more TD-like. In contrast to our second hypothesis, however, and unlike in TD individuals, fluid intelligence did not modulate accuracy on inference questions or regressions to context in participants with ADHD.

### Irony Processing and Comprehension by Adults with ADHD

A novel finding emerging from our study is that adults diagnosed with ADHD did not differ from neurotypical adults in their ability to interpret ironic utterances correctly. However, processing measures provided evidence that this came at a higher cost for adults with ADHD, complementing recent evidence showing difficulties with nonliteral meanings among adults with ADHD (Even-Simkin, 2024; Köder et al., 2024). Adults with ADHD produced significantly longer reading times for ironic compared to literal phrases, an effect which held despite irony inducing a significant processing cost also in TD readers, and despite ADHD readers exhibiting a significantly slower reading pace overall (Stern et al., 2024). That is, the significant interactions between Group and Phrase Type in both first pass gaze duration and total reading time of the Target Phrase Region suggest a specific irony processing cost for readers with ADHD.

Contrary to our expectations, there were no group differences in comprehension accuracy as measured by the explicit comprehension task. Specifically, while accuracy on the inference questions of the comprehension task in the irony condition was lower across both groups, individuals with ADHD were *not* less accurate than neurotypical individuals, as evidenced by the absence of a group effect. The processing cost observed for individuals with ADHD could therefore be the result of processing trade-offs, that is, investing more processing time in exchange for better accuracy, potentially as part of (subconscious) compensatory strategies. Research on general reading comprehension by individuals with ADHD has produced mixed findings, with some studies reporting comprehension problems and others not, with the results often depending on task selection (e.g., using open-ended questions; Avramovich & Yeari, 2024, or multiple-choice items; Segal, 2023), as well as other factors such as time constraints (for a review see Parks et al., 2022). Our findings on comprehension line up with those of Miranda et al. (2017), as we found that participants with ADHD performed equally well as TD participants on both inference and general content questions across both phrase types. This indicates that adults with ADHD do not exhibit impairments in general reading comprehension, inferential processes, or the ability to understand and attribute mental states to others.

Our finding that adults with ADHD comprehend ironic utterances equally well as adults without the disorder—albeit potentially through a slower, more cognitively demanding process—expands upon previous research indicating impaired irony comprehension in children with ADHD (Carruthers et al., 2022; Ludlow et al., 2017; Staikova et al., 2013). A possible explanation for the good performance of adults with ADHD could be that frequent exposure to ironical utterances over the years (cf. Gibbs, 2000) might have effectively trained these pragmatic skills. The finding that adults with ADHD are not impaired in understanding irony is important because it suggests that individuals with ADHD can fully engage in shared ironic exchanges in both professional and personal contexts. Such interactions are key in building and strengthening interpersonal relationships and fostering a sense of group membership (Attardo, 2000; Gibbs, 2000).

Nevertheless, further research is necessary to determine whether our results on irony comprehension during reading generalize to face-to-face interactions, where individuals must also consider additional ironic cues such as facial expressions, gestures, and tone of voice. The need to distribute attention across multiple sources of information could reveal potential challenges related to ADHD in irony comprehension in multi-modal settings.

### The Role of Executive Attention and ADHD in Processing

#### Fluid Intelligence

Regarding our second hypothesis, we did not find an effect of fluid intelligence in readers with ADHD—although those reported by Kyriacou and Köder (2025) for TD readers were still evident in model outputs. Intriguingly, we found that increased fluid intelligence led to better accuracy on inference questions for TD readers (regardless of phrase type), whereas the accuracy of readers with ADHD remained unaffected by fluid intelligence. This was despite the groups having equal fluid intelligence ability. As discussed in the Introduction, fluid intelligence benefits may depend on working memory capacity (Jastrzębski et al., 2018), especially for individuals with ADHD (Brydges et al., 2017, 2021). It is possible that the (albeit nonsignificant) lower working memory score of participants with ADHD relative to the control group prohibited a comparable fluid intelligence effect on accuracy and regression probability to context. This could indicate a minimum working memory requirement for effects of fluid intelligence to arise during irony processing. Future research on other populations with limited working memory capacity such as children or elderly people could further elucidate the interrelationship of fluid intelligence and working memory in deriving ironic meanings. Moreover, our findings also highlight potential limitations resulting from assuming that different groups of participants are truly “equal” when they are found to match on a given parameter, such as on a cognitive construct.

#### Working Memory

Working memory modulated processing in individuals with ADHD, and it specifically influenced the processing of irony: higher working memory made (first pass gaze and total) reading times as well as response times to inference questions comparable to those of TD individuals. In contrast, lower working memory led to significantly slower reading and response times compared to TD individuals. Moreover, although irony still induced significantly longer total reading times relative to literal language in individuals with ADHD and higher working memory capacity, there were no notable differences in the processing of irony compared to neurotypical individuals with equally high working memory capacity. It appears, therefore, that greater working memory in ADHD makes overall processing, as well as the processing of irony more efficient and more akin to that of TD individuals, thus minimizing the specific irony processing cost observed for ADHD.

Notably, in the irony condition, both groups exhibited increased regression probability to the Target Phrase Region as a function of *higher* working memory capacity. This means that participants with and without ADHD and greater working memory capacity were more likely to reread the target phrases when those were intended ironically. Despite the additional reading time imposed by those regressive refixations in the target phrase region, the total reading time of ironic utterances was still faster in readers with higher as opposed to lower working memory capacity, at least for participants with ADHD. We therefore argue that this again signals successful strategic processing, whereby individuals with higher working memory were better able to pinpoint and allocate additional processing resources to higher-demand items (i.e., ironic as opposed to literal phrases), without however sacrificing processing efficiency, as evidenced by the faster reading times.

Conversely, participants with lower working memory capacity produced fewer regressions to the target phrase region regardless of phrase type, thus indicating undifferentiated processing between higher- and lower-demand items. In the case of participants with ADHD, this was further accompanied by significantly slower reading times, as they seemingly adopted a more inflexible, all-in approach to processing regardless of actual processing demand. Notably, in TD participants, lower working memory did not significantly affect reading or response times, which begs the question of why participants with ADHD and lower working memory would exhibit worse performance relative to TD participants with equally low working memory.

Working memory is assumed to be responsible for the processing and manipulation of information during task performance, as well for the maintenance of attention to the task. Larger working memory capacity can bestow more efficient information processing, thus explaining why individuals with ADHD read (both phrase types) faster and responded to inference questions more quickly as working memory increased. At the same time, the lack of a similar effect in neurotypical readers suggests that working memory is particularly important in individuals with ADHD, potentially exacerbating or mitigating ADHD-related effects on processing speed (Miranda et al., 2017; Shanahan et al., 2006; Woods et al., 2002). It is also possible that the working memory task captured also some ADHD-specific variance, which was absent in the TD Group. To investigate this further, we retrospectively ran correlation tests between working memory scores and ADHD behavioral symptoms of inattention and hyperactivity, as calculated based on relevant Part B questions of the ASRS-v1.1 (Kessler et al., 2005). We found a significant negative correlation between working memory scores and hyperactivity symptoms for the ADHD Group (*r* = −.29, *t* = −2.12; *p* = .04), but not for the TD Group (*r* = −.03), whereas correlations between working memory scores and inattention symptoms were low and insignificant in both Groups (ADHD: *r* = −.17, *t* = −1.23, *p* = .22; TD: *r* = −.06, *t* = −0.045, *p* = .65). This suggests that, for individuals with ADHD, lower working memory scores were accompanied by more severe hyperactivity symptoms and higher working memory scores with less severe hyperactivity symptoms. Therefore, increased hyperactivity in tandem with lower working memory could have further interfered with participants’ ability to maintain focus to the task. As a result, this inflated reading and response times relative to both ADHD participants with less hyperactivity and higher working memory, and relative to TD participants with lower working memory who did not also experience hyperactivity.

Distractibility in ADHD is typically associated with inattention, as opposed to hyperactivity/impulsivity, which are instead associated with symptoms such as (physical) restlessness and fidgeting. Nevertheless, previous studies report that hyperactive and combined ADHD presentations exhibit worse performance than the inattentive subtype in the memory domain which requires attention (Dobson-Patterson et al., 2016; LeRoy et al., 2019). Our findings further suggest a potential interaction between working memory capacity and hyperactivity symptoms that affects reading behavior in individuals with ADHD. After all, physical restlessness in an experimental setting, where participants are required to sit still for extended periods of time, and without moving their head during the eye-tracking task, could have had a detrimental effect on the participants’ ability to focus on the reading task, thus directly affecting reading performance.

### Executive Attention in Irony Processing among TD and ADHD Individuals

As discussed in the Introduction, the effect of working memory on the processing of irony has produced mixed findings in the literature. Olkoniemi et al. (2016) and Kaakinen et al. (2014) found that greater working memory increased (re)reading time for ironic phrases during first pass reading time in TD participants, and argued that this signified earlier consideration of ironic meanings. In the present study, working memory only modulated the processing of irony by individuals with ADHD, although effects were seen in later eye-tracking measures (i.e., first pass gaze duration, total reading time, regression probability). Moreover, greater working memory was not found to increase reading times, but instead to reduce them, even though irony was still slower to read compared to literal language among readers with ADHD and higher working memory.

Additionally, previous findings indicated that readers with lower working memory were more likely to regress to ironic phrases, which was interpreted as evidence of compensatory strategies mobilized following comprehension difficulties (Olkoniemi et al., 2016; Olkoniemi, Johander, et al., 2019). In the present study we found the opposite: that is, regression probability to ironic phrases was higher with *greater* working memory in both TD and ADHD participants. We suggested that this reflects strategic processing as opposed to compensation mechanisms, since regressions were not accompanied by increased total reading times in the target phrase region.

The differences across studies could be due to variability in reading material complexity which placed different demands on working memory, or due to previous studies not incorporating a measure for fluid intelligence, thus leaving working memory to account for all processing variance. Additionally, differences in sample composition cannot be excluded. As our findings suggest a differential role of working memory and fluid intelligence in irony processing in adults with and without ADHD, replication from further studies could cast some additional light on this.

While we have discussed the potential impact of ADHD-specific factors that may have led to this differential effect on processing, and in particular the interaction of working memory with hyperactivity symptoms in ADHD, it is also possible that a lower working memory threshold is necessary for effects of working memory to appear. TD participants may have been well above this threshold, thus not allowing for (strong) working memory effects to emerge. It is worth reiterating that while groups did not significantly differ in their working memory scores, TD participants tended to score on average two points higher than ADHD participants. Therefore, while there were no visible deficits in executive attention for individuals with ADHD relative to TD participants, the lower scores in working memory may have been adequate for relevant effects to emerge in ADHD participants, whose inattention/hyperactivity symptoms might have further added to the cognitive load. It is also worth mentioning that the working memory task we used in the present study is specifically designed to cater for individual differences by removing influences from processing speed differences or speed-accuracy trade-offs (Draheim et al., 2021). This might explain why the performance of participants with ADHD was not significantly worse than that of TD participants, unlike what has been previously reported in the literature, where tasks were not adjusted for individual differences (Alderson et al., 2013; Schweitzer et al., 2006).

It seems, therefore, that both working memory and fluid intelligence are important to the processing of irony, but which construct will be most implicated might be determined by individual differences in these measures, as well as by additional cognitive demands imposed by factors such as hyperactivity in the case of ADHD. Furthermore, it is possible that a minimum working memory threshold is required for fluid intelligence to confer additional processing advantages, as problem-solving skills can be better accommodated within a larger working memory processing capacity. This might also explain why greater fluid intelligence did not improve accuracy on inference questions for ADHD participants, as it seemingly did for TD participants.

## Conclusion

In conclusion, our study is the first to provide evidence that irony comprehension is not impaired in adults with ADHD, as opposed to previous findings focusing on children with ADHD. However, despite a similar performance as controls in deriving ironic meanings, adults with ADHD exhibited specific processing costs for ironic utterances, revealing a potential speed-accuracy trade-off. This suggests the presence of compensatory strategies developed over time to cope with pragmatic language deficits. For individuals with ADHD, working memory capacity turned out to be an important predictor of pragmatic performance: not only did it speed up processing overall, working memory also mitigated the additional ADHD-related irony processing cost. In contrast, while fluid intelligence positively impacts inference accuracy in neurotypical readers, it does not appear to confer the same benefit in adults with ADHD, potentially due to lower working memory interfering with the efficiency of problem-solving skills, or due to additional challenges introduced by increased hyperactivity symptoms. Our results highlight the importance of assessing individual differences in cognition and behavior in both neurotypical and clinical populations, to better understand how these factors affect the processing of irony. Since pragmatic impairments linked to ADHD seem to become more subtle in adulthood, implicit measures such as eye-tracking should be employed, which are sensitive enough to reveal existing differences in language processing between adults with and without ADHD. Future research could investigate whether our findings for irony interpretation can be replicated for other types of non-literal language (e.g., metaphor, metonymy, and idiom), providing a more comprehensive picture of how ADHD affects communication abilities in adulthood.

## Appendix

**Appendix.** Final Model Outputs Across Eye-tracking Measures and Regions of Interest.

Predictors	Regression probability to context region				Regression probability to target phrase region				Accuracy on inference question				Response time to inference question			
	β	*SE*	*z*	*p*	β	*SE*	*z*	*p*	β	*SE*	*z*	*p*	β	*SE*	*z*	*p*
(Intercept)	.99	0.14	7.17	**<.001**	−1.29	0.11	−11.62	**<.001**	1.71	0.18	9.46	**<.001**	8.08	0.04	220.57	**<.001**
PhraseType [Irony]	.15	0.05	3.01	**.003**	.24	0.05	4.77	**<.001**	−.32	0.07	−4.57	**<.001**	.05	0.01	3.37	**.001**
Group [ADHD]	.57	0.13	4.41	**<.001**	.11	0.08	1.35	.176	−.18	0.1	−1.84	.065	0	0.02	0.13	.894
Fluid intelligence score	.01	0.01	1.44	.149	.01	0.01	1.38	.166	.01	0.01	1.71	.087	0	0	−0.85	.396
Working memory score	−.01	0.02	−0.64	.521	.01	0.01	0.54	.587	−.02	0.01	−1.31	.191	−.01	0	−2.18	**.029**
Trial index	−.03	0.01	−3.77	**<.001**	0	0.01	−0.02	.983	.03	0.01	3.05	**.002**	−.01	0	−10.67	**<.001**
PhraseType [Irony] × Group [ADHD]	−.02	0.05	−0.37	.708	.05	0.05	0.93	.352	.11	0.07	1.56	.119	0	0.01	0.21	.836
PhraseType [Irony] × Fluid intelligence score	0	0	−0.35	.728	−.01	0	−1.82	.069	.01	0.01	1.65	.098	0	0	0.04	.965
PhraseType [Irony] × Working memory score	0	0.01	0.76	.446	.02	0.01	3.03	**.002**	0	0.01	−0.06	.951	0	0	0.02	.981
Group [ADHD] × Fluid intelligence score	−.01	0.01	−0.61	.542	0	0.01	0.14	.888	−.02	0.01	−3.05	**.002**	0	0	1.94	.053
Group [ADHD] × Working memory score	0	0.02	0.13	.895	0	0.01	0.01	.993	.02	0.01	1.36	.173	−.01	0	−3.52	**<.001**
PhraseType [Irony] × Group [ADHD] × Fluid intelligence score	.01	0	2.23	**.025**	0	0	−0.25	.802	0	0.01	0.72	.471	0	0	1.38	.168
PhraseType [Irony] × Group [ADHD] × Working memory score	0	0.01	0.46	.645	0	0.01	0.31	.755	.01	0.01	0.87	.385	0	0	−0.69	.492
Random effects																
σ^2^	3.29				3.29				3.29				0.15			
τ_00_	1.42 _ppt_id_				0.45 _ppt_id_				0.51 _ppt_id_				0.05 _ppt_id_			
	0.06 _item_id_				0.12 _item_id_				0.52 _item_id_				0.02 _item_id_			
τ_11_													0.00 _ppt_id.PhraseType1_			
													0.00 _item_id.PhraseType1_			
ρ_01_													−0.14 _ppt_id_			
													−0.38 _item_id_			
ICC	0.31				0.15				0.24				0.32			
*N*	107 _ppt_id_				107 _ppt_id_				107 _ppt_id_				107 _ppt_id_			
	24 _item_id_				24 _item_id_				24 _item_id_				24 _item_id_			
Observations	2,568				2,563				2,148				2,565			
Marginal *R*^2^/conditional *R*^2^	.089/.372				.028/.173				.066/.289				.081/.378			
Predictors	First pass reading time of target phrase region				First pass gaze duration of target phrase region				Total reading time of target phrase region				First fixation duration of spillover region			
	*β*	*SE*	*t*	*p*	*β*	*SE*	*t*	*p*	*β*	*SE*	*t*	*p*	*β*	*SE*	*t*	*p*
(Intercept)	6.52	0.06	100.85	**<.001**	6.79	0.07	102.65	**<.001**	6.96	0.06	108.97	**<.001**	5.44	0.01	383.38	**<.001**
PhraseType [Irony]	.02	0.01	1.93	.054	.03	0.01	5.12	**<.001**	.07	0.01	5.57	**<.001**	.02	0.01	2.73	**.006**
Group [ADHD]	−.02	0.04	−0.57	.566	.08	0.03	2.61	**.009**	.09	0.03	2.9	**.004**	−.02	0.01	−1.53	.127
Fluid intelligence score	0	0	−0.86	.391	0	0	0.11	.914	0	0	0.68	.494	0	0	−0.51	.611
Working memory score	0	0	−1.04	.3	0	0	−1.3	.192	−.01	0	−1.55	.121	0	0	−0.91	.363
Trial index	0	0	0.35	.723	0	0	−4.69	**<.001**	−.01	0	−5.66	**<.001**	0	0	1.67	.096
PhraseType [Irony] × Group [ADHD]	.02	0.01	1.5	.135	.01	0.01	2.14	**.032**	.03	0.01	4.38	**<.001**	0	0.01	0.51	.609
PhraseType [Irony] × Fluid intelligence score	0	0	0.09	.931	0	0	1.71	.088	0	0	0.75	.455	0	0	−0.48	.633
PhraseType [Irony] × Working memory score	0	0	−0.43	.667	0	0	−0.96	.339	0	0	2.04	**.042**	0	0	0.77	.444
Group [ADHD] × Fluid intelligence score	0	0	1.2	.232	0	0	1.92	.055	0	0	1.6	.109	0	0	0.64	.52
Group [ADHD] × Working memory score	−.01	0	−1.9	.058	−.01	0	−2.43	**.015**	−.01	0	−2.07	**.038**	0	0	−1.36	.174
PhraseType [Irony] × Group [ADHD] × Fluid intelligence score	0	0	−1.37	.17	0	0	−0.79	.432	0	0	−1.25	.213	0	0	−0.28	.782
PhraseType [Irony] × Group [ADHD] × Working memory score	0	0	0.35	.724	0	0	−0.23	.818	0	0	1.37	.17	0	0	1.8	.073
Random effects																
σ^2^	0.39				0.12				0.14				0.09			
τ_00_	0.07 _ppt_id_				0.08 _ppt_id_				0.09 _ppt_id_				0.01 _ppt_id_			
	0.08 _item_id_				0.09 _item_id_				0.08 _item_id_				0.00 _item_id_			
τ_11_	0.01 _item_id.Group1_								0.00 _item_id.PhraseType1_				0.00 _item_id.PhraseType1_			
ρ_01_	0.27 _item_id_								0.03 _item_id_				−0.83 _item_id_			
ICC	0.3				0.59				0.54				0.13			
N	107 _ppt_id_				107 _ppt_id_				107 _ppt_id_				107 _ppt_id_			
	24 _item_id_				24 _item_id_				24 _item_id_				24 _item_id_			
Observations	2,544				2,559				2,561				2,538			
Marginal *R*^2^/Conditional *R*^2^	.013/.305				.056/.615				.071/.569				.012/.139			

Significant effects in bold.
